# Long Non-Coding RNAs: Key Regulators of Tumor Epithelial/Mesenchymal Plasticity and Cancer Stemness

**DOI:** 10.3390/cells14030227

**Published:** 2025-02-05

**Authors:** Yuan Yuan, Yun Tang, Zeng Fang, Jian Wen, Max S. Wicha, Ming Luo

**Affiliations:** 1 Department of Breast and Thyroid Surgery, Peking University Shenzhen Hospital, Shenzhen 518036, China; yuanyuanwork11@163.com (Y.Y.); ty98971106@163.com (Y.T.); fzncu609527@126.com (Z.F.); 2 Department of Breast Surgery, The Fourth Affiliated Hospital of China Medical University, Shengyang 110032, China; wenjian@cmu.edu.cn; 3 Division of Hematology & Oncology, Department of Internal Medicine, University of Michigan, Ann Arbor, MI 48109, USA

**Keywords:** lncRNAs, epithelial/mesenchymal plasticity, cancer stemness

## Abstract

Long non-coding RNAs (lncRNAs) are a class of non-coding RNA molecules with transcripts longer than 200 bp, which were initially thought to be noise from genomic transcription without biological function. However, since the discovery of H19 in 1980 and Xist in 1990, increasing evidence has shown that lncRNAs regulate gene expression at epigenetic, transcriptional, and post-transcriptional levels through specific regulatory actions and are involved in the development of cancer and other diseases. Despite many lncRNAs being expressed at lower levels than those of protein-coding genes with less sequence conservation across species, lncRNAs have become an intense area of RNA research. They exert diverse biological functions such as inducing chromatin remodeling, recruiting transcriptional machinery, acting as competitive endogenous RNAs for microRNAs, and modulating protein–protein interactions. Epithelial–mesenchymal transition (EMT) is a developmental process, associated with embryonic development, wound healing, and cancer progression. In the context of oncogenesis, the EMT program is transiently activated and confers migratory/invasive and cancer stem cell (CSC) properties to tumor cells, which are crucial for malignant progression, metastasis, and therapeutic resistance. Accumulating evidence has revealed that lncRNAs play crucial roles in the regulation of tumor epithelial/mesenchymal plasticity (EMP) and cancer stemness. Here, we summarize the emerging roles and molecular mechanisms of lncRNAs in regulating tumor cell EMP and their effects on tumor initiation and progression through regulation of CSCs. We also discuss the potential of lncRNAs as diagnostic and prognostic biomarkers and therapeutic targets.

## 1. Introduction

Long non-coding RNAs (lncRNAs) are described as long transcripts (more than 200 nucleotides), lacking protein-coding potential. Recent advances in sequencing technologies have revealed that only ~2% of the human genome codes for proteins, while the remainder is composed of non-coding regions that were once believed to be “junk DNA”. In fact, lncRNAs constitute almost 68% of total RNA transcripts in humans [1], where they are derived from different genomic regions, including intergenic regions (lincRNA), enhancer (elncRNA), promoter (plncRNA), exons, and introns [2]. LncRNAs participate in the regulation of gene expression at multiple levels via interacting with DNA, RNA, and proteins, and their intracellular distribution is usually associated with their functions. By interacting with chromatin modifiers and transcription and splicing factors, nuclear lncRNAs are involved in modulating the structure and accessibility of chromatin and regulating the transcription and alternative splicing of RNA [3,4,5], while cytoplasmic lncRNAs are mainly involved in the regulation of mRNA and rRNA function [6,7,8], protein translation [9,10], as well as protein–protein interactions [11]. It is well established that lncRNAs participate in various physiological processes, such as cell differentiation [12,13], programmed cell death [14], autophagy [15], immunity [16], and metabolism [17]. Moreover, they are also implicated in the pathogenesis of numerous diseases, including cardiovascular disease [18], osteoarthritis [19], and cancer [20]. Abundant lncRNAs have been reported to be dysregulated in multiple carcinomas, where their expression levels are associated with clinicopathologic features and prognosis, indicating important roles of lncRNAs in tumor development. As a result, these RNA molecules hold enormous potential as therapeutic targets. Accumulating evidence shows that lncRNAs play crucial roles in the regulation of cancer stem cells (CSCs) [21,22,23]. These cells are characterized by the capacity for self-renewal to replenish themselves as well as differentiation to produce diverse tumor cell populations constituting the tumor bulk. The existence of CSCs has been reported in various carcinomas, including leukemia [24], breast cancer (BC) [25], gastric cancer (GC) [26], non-small cell lung cancer (NSCLC) [27], pancreatic cancer (PC) [28], and colon cancer [29], where they are responsible for tumor initiation, growth, metastasis, and therapeutic resistance. LncRNAs are reported to modulate CSC self-renewal [8,30,31] and differentiation [32,33], as well as asymmetric self-renewal [34]. In addition, although CSCs may be derived from tissue stem cells or progenitor cells with dysregulated self-renewal, published studies have revealed a link between EMT and acquisition of cancer cell stemness [35,36]. EMT is a dynamic process in which epithelial cells lose cell–cell junctions and apicobasal polarity and acquire mesenchymal characteristics, leading to increased migratory and invasive capacities. EMT and its reverse process, mesenchymal-to-epithelial transition (MET) play crucial roles in tumor metastasis. EMT facilitates the dissemination of cancer cells from primary tumors to remote tissues and endows the disseminated tumor cells (DTCs) with increased ability to survive environmental stress such as nutrient deprivation, hypoxia, oxidative stress, and therapeutic pressure [35,37,38]. In contrast, MET facilitates the proliferation and colonization of DTCs in distant organs to form clinically significant metastatic lesions [39,40,41]. The EMT/MET plasticity of tumor cells is tightly controlled by a core set of EMT transcription factors (EMT-TFs), which constitute three distinct protein families: the SNAIL family of zinc-finger TFs including SNAI1 (also known as SNAIL or SNAIL1) and SNAI2 (also known as SLUG), the zinc-finger E-box-binding homeobox TFs including ZEB1 and ZEB2, and the basic helix-loop-helix TFs including TWIST1 and TWIST2 [42]. These EMT-TFs, through downregulation of E-cadherin and upregulation of N-cadherin and vimentin expression, promote EMT and facilitate cancer progression and metastasis [42]. LncRNAs regulate tumor cell epithelial/mesenchymal plasticity (EMP) by regulating EMT-TFs and EMT-associated signaling pathways such as TGFβ/SMAD, WNT/β-catenin, Notch, and receptor tyrosine kinase signaling pathways [43,44,45,46]. Here, we describe some essential points on the present knowledge regarding the roles and mechanisms of lncRNAs in regulating tumor cell EMP and cancer stemness, as well as the impact of these lncRNAs on tumor initiation and progression. Furthermore, we discuss the diagnostic and therapeutic implications of these tumor-associated lncRNAs.

## 2. The Major Functions of lncRNAs

LncRNAs carry out their biological functions by interacting with DNA, RNA, and proteins. Several modes of action have been reported: (1) scaffold—lncRNAs can directly bind to proteins and affect their affinity to interacting partners, thus regulating protein–protein interactions [47]; (2) sponge—lncRNAs can competitively bind to microRNAs (miRNAs) through short complementary sequences, thereby suppressing miRNA binding to target mRNAs [48,49,50,51]; (3) guide—lncRNAs can interact with diverse transcription factors and chromatin modifiers and recruit them to the genomic regions where they operate [52,53]; (4) decoy—lncRNAs can sequester transcription factors, chromatin modifiers, and other functional proteins, thereby inhibiting their functions [54].

### 2.1. LncRNA-Mediated Regulation of Gene Expression at Epigenetic and Transcriptional Levels

**Figure 1:**
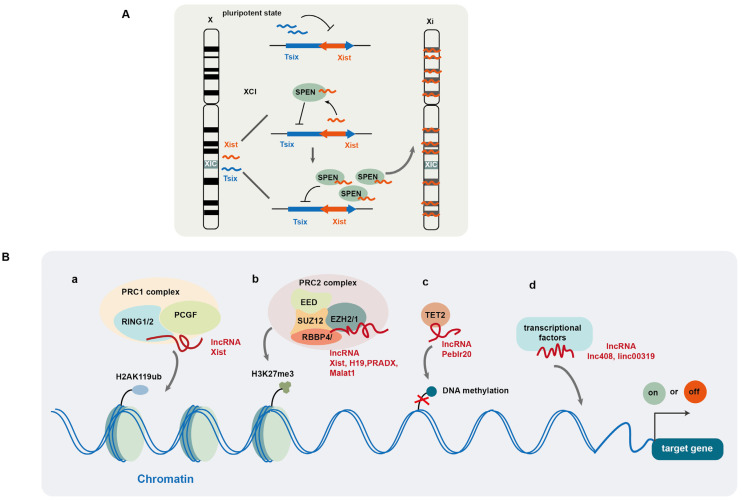
LncRNA-mediated regulation of gene expression at epigenetic and transcriptional levels. (**A**) LncRNA Xist plays a key role in X chromosome inactivation (XCI) during early embryonic development in female mammals; (**B**) lncRNAs regulate gene expression by modulating histone (**a**,**b**) and DNA (**c**) modification and recruiting transcription factors (**d**).

LncRNA Xist, which is known to play a key role in X chromosome inactivation (XCI), silences gene expression in one of the X chromosomes during early embryonic development in female mammals [55]. Its encoding gene *Xist* is located at a specific locus of the X chromosome, called the X inactivation center (XIC) [56,57] (Figure 1A). During early embryonic development in female mammals, Xist controls XCI initiation. Recent studies have revealed that the RNA-binding protein SPEN participates in XCI initiation by regulating the expression of Xist and its antisense lncRNA Tsix [58]. In the pluripotent state, Tsix is expressed from both X chromosomes and suppresses the expression of Xist in embryonic stem cells (Figure 1A). In late embryonic stages, XCI is triggered by the monoallelic expression of Xist in the future inactive X chromosome (Xi), where Xist binds to the SPEN protein via its repeat A region which then recruits SPEN to the Tsix promoter where it suppresses Tsix expression. SPEN-mediated Tsix repression induces the upregulation and accumulation of Xist, which then spreads along the future Xi and silences X-linked genes in cis at the entire Xi chromosome (Figure 1A). Once XCI is initiated, Xist wraps the Xi chromosome and recruits multiple silencing factors (such as histone deacetylase and chromatin remodelers), which induce a cascade of conformation changes in chromatin, including HDAC3-mediated histone deacetylation, chromatin compaction, exclusion of RNA polymerase II, removal of markers for active X chromosome (Xa), and accumulation of Xi markers [59]. SPEN is crucial for Xist-mediated gene silencing by bridging Xist to the silencing factors [60]. SPEN protein contains four N-terminal RNA recognition motifs (RRM) and a C-terminal SPOC domain. Through its RRM domain, SPEN directly interacts with Xist, while through the SPOC domain, SPEN binds to diverse proteins to form a large macromolecular complex. A number of proteins interact with the SPOC domain, including NcoR/SMRT, the NuRD complex, m6A RNA methylation machinery, and RNA polymerase II, all of which are implicated in XCI establishment [60]. In normal mammary epithelial cells, the maintenance of XCI appears to counteract malignant transformation as Xist deficiency impairs human mammary stem cell (MaSC) differentiation and facilitates tumorigenicity [32]. Mechanistically, Xist loss results in epigenetic changes and partial transcriptional reactivation of X-linked genes on Xi, including the gene-coding mediator subunit *MED14.* This overdosage of *MED14* results in hyperactivation of the MaSC enhancer landscape, making stem cell differentiation less favorable and promoting tumorigenesis [32]. However, in transformed tumor cells, Xist appears to have a role in helping to maintain CSCs as histone deacetylase inhibitor (HDACi) Abexinostat induces the differentiation of CSCs in breast cancer with low Xist expression, and Xist has been identified as a biomarker for predicting the response to HDACi [61].

The polycomb repressive complexes (PRCs), including PRC1 and PRC2, are well known to regulate histone post-translational modification. PRC1 exhibits E3 ubiquitin ligase activity and regulates mono-ubiquitination of histone 2A at Lys119 (H2AK119ub) [62]. Its catalytic core consists of RING1 or RING2, as well as one of polycomb group’s RING finger (PCGF) proteins. PRC2 induces monomethylation, dimethylation, and trimethylation of Lys27 (H3K27me, H3K27me2, H3K27me3) [63]. Its core comprises an enhancer of Zeste2/1 (EZH2/1), embryonic ectoderm development (EED), Suppressor of Zeste (SUZ12), and retinoblastoma-binding protein 4/7 (RBBP4/7) [62,64]. Among these proteins, EZH2 exhibits methyltransferase activity. Several lncRNAs have been reported to interact with PRCs and recruit them to chromatin to regulate histone modification (Figure 1B(a,b)) [52,65,66]. It is documented that PRC1 and PRC2 are recruited to chromatin by Xist via its B-repeat and A-repeat elements, respectively, and PRC-induced histone modification is required for Xist-mediated chromosome silencing [67,68].

LncRNA H19, by binding to EZH2 and facilitating PRC2 complex formation, induces H3K27me3 or H3K4me3 modification on the genes associated with the androgen receptor pathway in neuroendocrine prostate cancer [52]. LncRNA PRADX also binds to EZH2 and recruits the PRC2/DDX5 complex to the UBXN1 gene and induces H3K27me3 at its promoter, leading to UBXN1 suppression [65]. The lncRNA SCIRT (Stem Cell Inhibitory RNA Transcript) alters the affinity of EZH2 for its protein-binding partners to regulate cancer cell state transitions. Specifically, SCIRT interacts with EZH2 to increase EZH2 affinity to FOXM1 (a transcriptional regulator), which antagonizes EZH2-mediated suppression of cell-cycle gene expression, thereby promoting differentiation. However, for CSCs or tumor-initiating cells, FOXM1 is absent and SCIRT antagonizes EZH2 and SOX2 activity, balancing toward repression of self-renewal to restrain tumorigenesis [5]. In addition, the lncRNA HOX transcript antisense RNA (HOTAIR) recruits lysine-specific demethylase 1 (LSD1) and decreases H3K9me2 level at the promoter of MAPK1, leading to elevated MAPK1 expression in liver cancer [69]. The lncRNA SNHG29, which acts as a tumor suppressor in acute myeloid leukemia (AML), interacts with E1A binding protein p300 (EP300), a histone acetyltransferase, and decreases the enrichment of EP300 at the promoters of AML-related genes, thus suppressing the expression of these genes by modulating EP300-mediated histone acetylation modification [70].

In addition to modification of histone proteins, lncRNAs are also involved in epigenetic DNA modification to regulate gene transcriptional activity [71,72]. For example, lncRNA Peblr20 has been reported to promote pluripotent reprogramming in induced pluripotent stem cells (iPSCs). Peblr20 recruits TET2 demethylase to the Pou5F1 enhancer locus, thus activating Pou5F1 enhancer gene transcription by promoting DNA demethylation [71] (Figure 1B(c)). LncRNA AC006129.1 promotes the recruitment of DNA methyltransferases to the promoter of transcriptional repressor Capicua (CIC) and induces DNA methylation, leading to the downregulation of CIC protein [72]. LncRNAs also mediate the recruitment of transcriptional factors to promote gene expression (Figure 1 B(d)) [4,73]. For example, Linc00319 upregulates the expression of high-mobility group box3 (HMGB3) by recruiting E2F transcription factor 1 (E2F1) in laryngeal squamous cell carcinoma (LSCC) [73], while lncRNA408 induces chibby1 (CBY1) downregulation in breast cancer stem cells (BCSCs) by mediating the transcription factor SP3 to its promoter, thereby suppressing CBY1 transcription [4]. Therefore, by mediating chromatin (histone and DNA) modification and recruitment of transcriptional factors, lncRNAs can efficiently modulate gene expression at the transcriptional level.

### 2.2. LncRNA-Mediated Regulation of Gene Expression at Post-Transcriptional Levels

**Figure 2:**
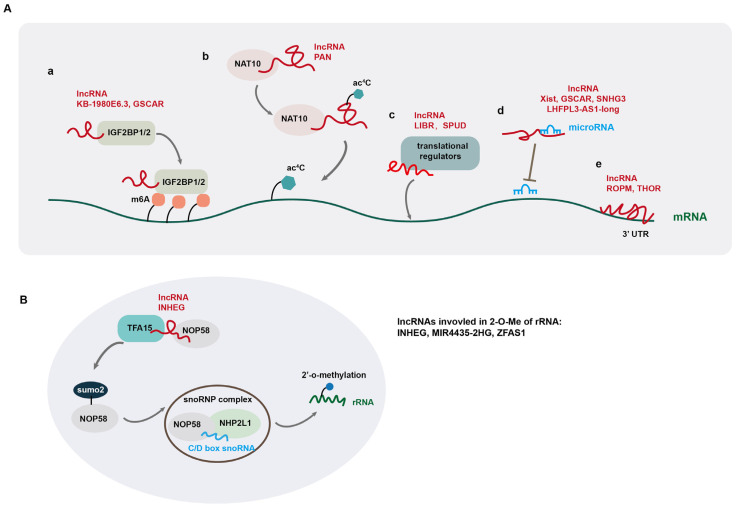
LncRNA-mediated regulation of gene expression at post-transcriptional levels. (**A**) LncRNAs regulate gene expression by modulating mRNA modification (**a**,**b**), recruiting translational regulators (**c**), sponging miRNA (**d**), and directly binding to mRNA (**e**); (**B**) lncRNAs are involved in the regulation of 2’-O-methylation (2-O-Me) of ribosomal RNA (rRNA).

At the post-transcriptional level, lncRNAs have been reported to regulate mRNA stability and alternative splicing and translation via different mechanisms. LncRNA KB-1980E6.3 increases the stability of c-myc mRNA by recruiting insulin-like growth factor 2 mRNA-binding protein 1 (IGF2BP1), a N6-methyladenosine (m6A) RNA reader, to the mRNA [74] (Figure 2A(a)). IGF2BP1 recognizes the m6A-modified RNA and stabilizes it by interacting with the coding region instability determinant (CRD) within the mRNA. Other IGF2BP proteins (IGF2BF2 and IGF2BP3) also affect mRNA stabilization via the recognition of RNA m6A modification [75]. LncRNA GSCAR promotes the stability of SOX2 mRNA by mediating the formation of a complex containing IGF2BP2 and DHX9 [11]. In addition, Zhang et al. reported that lncRNA FOXM1-AS facilitates the interaction between FOXM1 pre-mRNA and α-ketoglutarate-dependent dioxygenase alkB homolog 5 (ALKBH5), which is an RNA m6A demethylase, and induces the demethylation of FOXM1 pre-mRNA. This ALKBH5-mediated RNA demethylation facilitates the binding of HuR protein to the unmethylated 3’ untranslated region (UTR) of FOXM1 pre-mRNA, leading to increased RNA stability and upregulation of FOXM1 [76]. Moreover, lncRNA PAN is involved in regulating N4-acetylcytidine (ac^4^C) modification of mRNA, by interacting with N-acetyltransferase 10 (NAT10), which catalyzes RNA acetylation at its cytidine N^4^ position [77] (Figure 2A(b)). NAT10 induces ac^4^C modification of PAN to increase its stability. Subsequently, the acetylated PAN recruits NAT10 to IFI16 mRNA to promote its ac^4^C modification, leading to increased IFI16 mRNA stability and translation [77]. LncRNA DIO3OS interacts with polypyrimidine tract binding protein 1 (PTBP1), a suppressor of alternative splicing, to stabilize the mRNA of lactate dehydrogenase A and upregulate its expression by protecting the integrity of its 3’ UTR [78].

Besides regulating mRNA stability, lncRNAs play key roles in regulating mRNA alternative splicing, which in turn affects tumorigenesis and progression. For instance, lncRNA CTC-490G23.2 is implicated in the regulation of mRNA alternative splicing of CD44, a mesenchymal CSC marker in multiple cancer types [79]. In esophageal squamous cell carcinoma (ESCC), NAT10-mediated ac4C modification of CTC-490G23.2 increases its stability, which functions as a scaffold to promote the interaction between polypyrimidine tract-binding protein1 (PTBP1) and CD44 pre-mRNA. By facilitating PTBP1-mediated alternative splicing of CD44 mRNA, CTC-490G23.2 upregulates the expression of CD44 variant isoform CD44v (8-10), which promotes cancer metastasis [79]. In hepatocellular carcinoma (HCC), LINC01089, a super enhancer-driven lncRNA, interacts with heterogeneous nuclear ribonucleoprotein M (hnRNPM) and promotes hnRNPM-mediated skipping of DIAPH3 exon 3, which contains an important m6A-modification site recognized by the m6A reader IGF2BP3 to stabilize DIAPH3 mRNA. Such alternative splicing of DIAPH3 promotes ERK signaling, EMT, and invasion and metastasis of HCC cells [3]. In gastric cancer (GC), lncRNA CRNDE directly binds to splicing protein SRSF6 to reduce its protein stability, which in turn reduces PICALM alternative splicing, triggering a significant switch from the short to long isoform to induce attenuated chemoresistance [80]. Similarly, LINC01852 suppresses SRSF5-mediated alternative splicing of PKM, which in turn inhibits the tumorigenesis and chemoresistance of colorectal cancer cells [81]. In addition to regulating alternative splicing of mRNAs, lncRNAs are often alternatively spliced, producing isoforms with divergent biological functions in cancer development [82]. 

In addition to regulating mRNA stability and alternative splicing, some lncRNAs are reported to regulate RNA translation by recruiting translational regulators to mRNA (Figure 2A(c)). For example, lncRNA LIBR suppresses BRD4 translation by recruiting RCK, a translation repressor to BRD4 mRNA, and blocking the binding of BRD4 mRNA to polysomes [34], while lncRNA SPUD is involved in the regulation of p21 translation by interacting with two competitive translational regulators (the activator CUGBP1 and inhibitor calreticulin) and modulating their association with p21 mRNA under DNA-damaging conditions [9].

Another mode of lncRNA post-transcriptional regulation is the so-called “sponge function” by acting as competing endogenous RNAs (ceRNAs) for microRNA (miRNA). These lncRNAs, via direct association with their cognate miRNAs, block the association of miRNAs with their target mRNAs, thus eliminating the suppressive effects of miRNAs on gene expression [11,48,49,80,81,82,83,84] (Figure 2A(d)). For example, lncRNA TUG1 promotes the self-renewal of glioma stem cells (GSCs) by sponging miR-145 in the cytoplasm and recruiting PRC2 complex to repress differentiation genes by locus-specific methylation of histone H3K27 via YY1-binding activity in the nucleus [85]. LncRNA Xist upregulates IL-6 expression by sponging tumor suppressor miRNA let-7a-2-3p, leading to increased STAT3 activation and an increase in ALDH^+^ breast CSCs [48]. Several lncRNAs positively regulate gene expression by directly binding to 3’ untranslated region (UTR) of mRNA, which promotes mRNA stability (Figure 2A(e)) [86,87]. In this regard, lncROPM promotes the stability of PLA2G16 mRNA by binding directly to its 3’-UTR, leading to elevated PLA2G16 expression in breast cancer [86]. LncRNA THOR also upregulates SOX9 expression by directly binding to the 3’UTR of its mRNA [87].

In addition, several studies have documented that lncRNAs regulate 2’-O-methylation (2-O-Me) of ribosomal RNA (rRNA), thereby affecting protein translation [7,8,88]. 2’-O-Me modification in rRNA is carried out by ribonucleoprotein (snoRNP) complexes, containing methyltransferase fibrillarin (FBL), small nucleolar RNAs (snoRNAs) from the C/D box snoRNA family, RNA-binding proteins NHP2L1, and the core proteins NOP56 and NOP58. The 2’-O-Me modification of rRNA promotes the stability of rRNA scaffolds to affect the accuracy and efficiency of translation [89]. LncRNA INHEG is highly expressed in glioma stem cells (GSCs) and promotes GSC self-renewal and tumorigenicity through control of rRNA 2’-O-methylation. Mechanistically, INHEG promotes the interaction between NOP58 and TAF15, a SUMO2 E3 ligase, leading to the SUMOylating of NOP58, which is crucial for snoRNP complex formation and rRNA 2’-O-methylation, leading to elevated translation of oncogenic proteins in glioma [8] (Figure 2B). LncRNA MIR4435-2HG is also reported to regulate rRNA 2’-O-Me modification in hepatocellular carcinoma (HCC) [7]. MIR4435-2HG directly interacts with IGF2BP1 and NOP58. IGF2BP1 increases MIR4435-2HG level via m6A modification. Moreover, MIR4435-2HG enhances rRNA 2’-O-Me modification by protecting NOP58 protein from degradation, thereby facilitating oncogene translation in an internal ribosome entry site (IRES)-dependent manner in HCC [7].

### 2.3. LncRNA-Mediated Regulation of Gene Expression at Post-Translational Level

**Figure 3:**
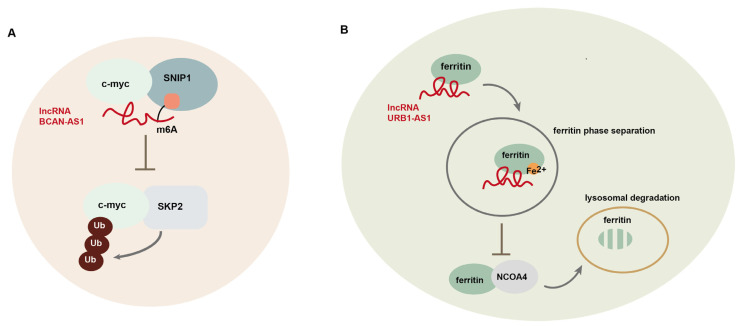
LncRNA-mediated regulation of gene expression at post-translational level. (**A**) LncRNA BCAN-AS1 participates in the regulation of c-myc ubiquitination and degradation in pancreatic ductal adenocarcinoma (PDAC); (**B**) lncRNA URB1-AS1 is involved in the regulation of sorafenib-induced ferroptosis in hepatocellular carcinoma (HCC) through modulating ferritin degradation.

LncRNAs also function as scaffolds to regulate protein–protein interaction. Moreover, they affect protein stability through the regulation of protein modification [10,90,91]. For example, lncRNA BCAN-AS1 is involved in the regulation of c-myc ubiquitination and degradation in pancreatic ductal adenocarcinoma (PDAC) [10]. BCAN-AS1 is m6A modified by an m6A methyltransferase METTL3, and its m6A modification is recognized by Smad nuclear-interacting protein1 (SNIP1), an m6A mediator. This lncRNA functions as a scaffold to strengthen the interaction between SNIP1 and c-myc, thereby suppressing the ubiquitination of c-Myc, mediated by S phase kinase-associated protein 2 (SKP2), a SKP1-CUL1-F-box protein (SCF) E3 ubiquitin ligase (Figure 3A). LncRNA PVT-1 induces the degradation of tumor suppressor complex 2 (TSC2) in osteosarcoma [91]. PVT-1 interacts with phosphatidylinositol 3-kinase catalytic subunit type 3 (Vsp34) and induces its SUMOylation, leading to TSC2 ubiquitination. LncRNA NRON binds to murine double minute 2 (MDM2) and MDMX, respectively, and induces heterodimerization between them, thereby enhancing the E3 ligase activity of MDM2 toward tumor suppressor substrates and promoting tumorigenesis in breast cancer [92]. In addition, lncRNA URB1-AS1 is involved in the regulation of sorafenib-induced ferroptosis in HCC through modulating ferritin degradation [93]. By interacting with ferritin to induce ferritin phase separation, URB1-AS1 inhibits lysosomal degradation of ferritin mediated by nuclear receptor coactivator 4 (NCOA4), which transports ferritin to lysosomes (Figure 3B). Additionally, lncRNA DARS1-AS1 has been shown to control post-transcriptional circuitry, promoting glioblastoma (GBM) stem cell-like cells. Mechanistically, DARS1-AS1 directly interacts with the YBX1 protein to promote target mRNA binding and stabilization, forming a mixed transcriptional/post-transcriptional feed-forward loop to upregulate expression of the key regulators of cell-cycle progression, including E2F1 and CCND1 [94]. LncBRM (gene symbol *LINCR-0003*) is highly expressed in liver CSCs, which associate with Brahma (BRM) to initiate the BRG1/BRM switch and the BRG1-embedded BAF complex formation, triggering the activation of YAP1 signaling [95]. LncRNA FAISL, by interacting with focal adhesion kinase (FAK) at its C-terminus domain, inhibits Calpain 2-mediated proteolysis of FAK, thereby promoting FAK-mediated tumor progression and metastasis [96].

## 3. The Regulatory Roles of lncRNAs in EMP and CSCs

EMT is a dynamic process, during which epithelial cells progressively lose their epithelial features, and at the same time, acquire mesenchymal characteristics. Normal epithelial cells are packed closely together by cell–cell junctions including tight junctions, gap junctions, and desmosomes and are attached to the basement membrane via hemidesmosomes [35]. These cells exhibit apical–basal polarity and express epithelial-associated genes. During EMT, epithelial cells lose cell–cell junctions and hemidesmosomes and acquire mesenchymal characteristics, accompanied by increased motility. Conversely, cells that have undergone EMT can be induced to repress mesenchymal-associated genes and regain epithelial characteristics, accompanied by the acquisition of front–back polarity. These dynamic EMP processes are closely regulated by the tissue microenvironment and play important roles in embryonic development, wound healing, and carcinoma development [97].

In the context of tumorigenesis, the EMT program is transiently activated and generates a spectrum of cells within intermediate states comprising a spectrum between the fully differentiated epithelial and mesenchymal cells [35]. These cells are also described as “quasi-mesenchymal cells” or “hybrid E/M cells” with increased CSC activity [35,98]. By activating EMT, tumor cells are conferred increased abilities for tumor initiation, invasion, and metastasis, as well as chemoresistance. However, sustained overexpression of EMT-associated transcription factors, such as TWIST1 and SNAI1, induces extreme EMT that suppresses CSC activity [35,99,100]. It was reported that induced expression of SNAI1 in combination with knockout of ZEB1 in basal breast cancer cells can push the cells into a quasi-mesenchymal state conferring them with high tumorigenic capacity [101], suggesting that induction to a quasi-mesenchymal state is crucial for maintaining cancer stemness. The EMT and MET-induced tumor plasticity is required for the dissemination of cancer cells from the primary tumors as well as the formation of metastatic colonies in distant tissues [35]. The tumor cells in a highly mesenchymal state can disseminate from the primary tumor, invade the circulatory systems, and seed distant organs. However, they fail to form metastatic colonies, which may be attributed to their limited proliferative capacity. On the other hand, although primary tumor cells in a full epithelial state can undergo proliferation, they exhibit less migratory and invasive capacities and may fail to initiate tumor formation in the absence of neighboring cancer cells [35,102]. The quasi-mesenchymal cells exhibit highly metastatic ability. These cells acquire invasion and migration capacities via a transient EMT program to disseminate to a distant tissue, where they undergo the MET program to regain full proliferative capacity and initiate the outgrowth of metastatic colonies.

Accumulating lines of evidence have revealed the pivotal roles of lncRNAs in regulating E/M plasticity and CSC activity during tumor development. Topel et al. demonstrated that lncRNA HOTAIR facilitates the hybrid E/M phenotype in hepatocellular carcinoma (HCC) cells via suppressing c-Met signaling [45]. HOTAIR inhibits the c-Met-induced fully mesenchymal phenotype and participates in activating metastasis-associated processes, such as cell adhesion, proliferation, colony formation, and motility [45]. LncRNA H19 regulates E/M plasticity in breast cancer via sponging miR-200b/c and let-7b [103]. Specifically, H19 promotes metastatic initiation via regulation of let-7b/CYTH3 and colonization via the miR-200b/c/Git2 axis [103]. In castration-resistant neuroendocrine (NE) prostate cancer (PCa), H19 regulates PCa lineage plasticity by driving a bidirectional cell identity of NE phenotype, with H19 overexpression promoting the castration-resistant NE phenotype, while H19 knockdown promotes the luminal type of PCa, which is sensitive to androgen deprivation therapy [52]. LncRNA MALAT1 acts as a ceRNA of miR-141-5p and elevated expression of MALAT1 promotes EMT of pancreatic cancer cells through the miR-141-5p-TGFβ-TGFβR1/TGTβR2 axis [104]. In another study, lncRNA HOTTIP was reported to modulate EMT and cell invasion as well as chemoresistance in pancreatic cancer by regulating HOXA13 [105]. 

LncRNAs also participate in the regulation of CSC activities by inducing EMT and CSC marker expression. For example, lncRNA HOTTIP mediates HOXA9 expression to enhance Wnt/β–catenin pathway activation by binding to WDR5 in pancreatic cancer stem cells (PCSCs), leading to upregulated expression of CSC markers (CD44, CD133, and ALDH1) as well as stem-associated factors (SOX2, OCT4, LIN28, and NANOG) [106]. Linc-DYNC2H1-4 acts as a ceRNA for miR-145 and facilitates the expression of its target genes, including ZEB1, a key regulator of EMT, as well as CSC markers SOX2, OCT4, NANOG, and Lin28, thereby promoting EMT and CSC activity in pancreatic cancer [107]. HOTTIP was also demonstrated to act as a ceRNA by sponging miR-574-5p, which promotes the progression of small cell lung cancer (SCLC) by affecting the expression of polycomb group protein EZH1 [108]. 

## 4. LncRNAs in Regulation of EMP-Associated Signaling

EMT is regulated by diverse EMT-inducing transcription factors (EMT-TFs), including the zinc-finger E-box-binging homeobox (ZEB factors) (ZEB1/2), TWIST family proteins (TWIST1/2), and SNAIL family proteins (SNAIL and SLUG) [98]. These EMT-TFs induce mesenchymal-associated genes and suppress epithelial-associated genes to promote the acquisition of a mesenchymal state. For example, ZEB1 promotes the expression of N-cadherin and vimentin, while repressing the expression of E-cadherin [109]. EMT-TFs also induce the expression of matrix metalloproteinases (MMPs), which mediate the degradation of extracellular matrix and facilitate EMT-induced invasion and metastasis [107,110].

Increasing evidence suggests that lncRNAs are associated with EMT by modulating the expression of EMT-TFs. Several lncRNAs have been reported to regulate the expression of EMT-TFs by sponging EMT-associated miRNAs, including the miRNAs belonging to the miR-200 family, which suppress the expression of ZEB1/2, TWIST, and SNAIL [108,111,112]. In gastric cancer (GC), lncRNA MAGI2-AS3 promotes ZEB1 expression and EMT by sponging miR-141/200a, thus facilitating cellular migration and invasion [108]. In bladder cancer, lncRNA SOX2OT acts as a ceRNA of miR-200c and promotes EMT, invasion, and bladder cancer stem cell activity through increased SOX2 expression [111]. LncRNA MIR200CHG promotes the expression of miR-200c and miR-141, repressing EMT and metastasis [112], while LINC00501 upregulates SLUG expression by recruiting hnRNPR protein to its promoter, facilitating EMT and angiogenesis in GC [113]. LncRNAs also modulate the expression of proteins associated with epithelial and mesenchymal properties, by regulating the mRNA stability. For instance, SNHG8, an lncRNA downregulated by ZEB1, promotes the stabilization of CDH1 mRNA [114].

### 4.1. TGFβ /Smad Pathway

**Figure 4:**
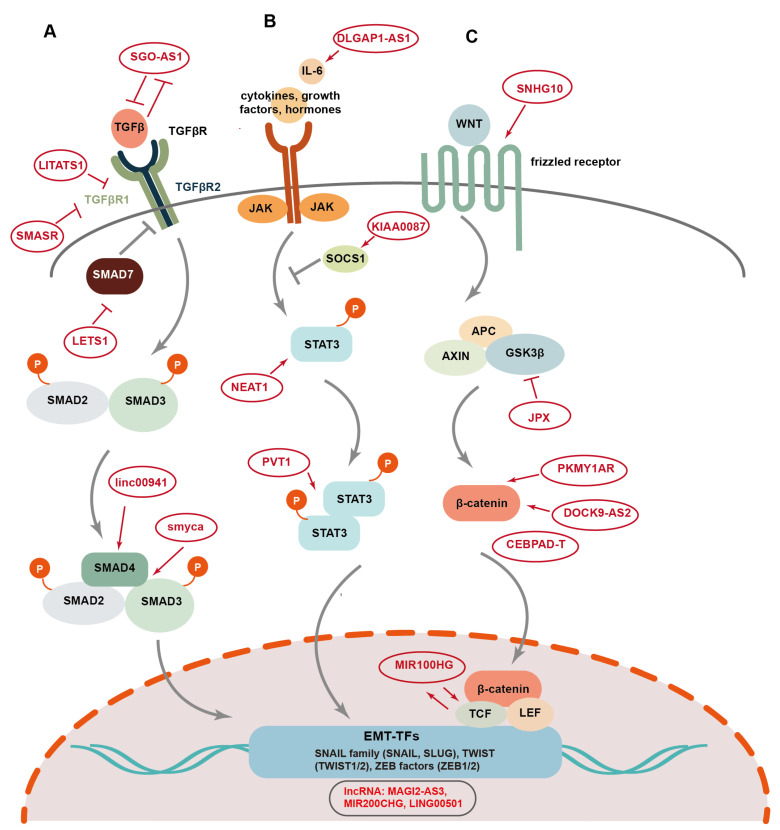
The EMP-associated signaling pathways regulated by lncRNAs in tumorigenesis and progression. LncRNAs regulate several EMP-associated signaling pathways, including TGF-β/Smad (**A**), STAT3 (**B**), and Wnt pathways (**C**), thus modulating EMT-mediated tumor development.

The transforming growth factor β (TGFβ) acts as a central node to mediate extracellular matrix (ECM) signaling by activating the expression of EMT-associated lncRNAs, which in turn regulate TGFβ expression and TGFβ-induced EMT [47,115,116,117,118,119] (Figure 4A). The TGFβ/SMAD signaling pathway is activated by the binding of the TGFβ ligand and its cognate receptor TGFβR, including TGFβ receptor type 1 (TGFβR1) and TGFβ receptor type 2 (TGFβR2), resulting in the phosphorylation of SMAD2 and SMAD3, which form a trimeric complex with SMAD4. Subsequently, the SMAD complex translocates into the nucleus to induce the expression of diverse EMT-associated genes, including EMT-TFs [98]. In contrast to SMAD4, SMAD7 inhibits TGFβ signaling by promoting TGFβR1 polyubiquitination and degradation [120]. Furthermore, TGFβ expression is in turn regulated by the EMT-TFs, forming a positive feedback loop to enhance EMT. A number of lncRNAs have been documented to either activate or inhibit TGFβ/SMAD signaling. For example, lncRNA SGO1-AS1 has been shown to suppress EMT and tumor metastasis in GC by inhibiting TGFβ signaling [121]. SGO1-AS1 promotes the degradation of TGFβ1/2 mRNA by competitively binding to the PTBP1 protein, promoting the downregulation of TGFβ expression [122]. On the other hand, TGFβ counteracts SGO1-AS1 through ZEB1, thereby forming a negative feedback loop with SGO1-AS1 [121]. Contrary to SGO1-AS1, lncRNA LITATS1 is activated by TGFβ/SMAD signaling, which in turn suppresses TGFβ-induced EMT and migration of cancer cells in non-small cell lung cancer [115]. LITATS1 interacts with TGFβR1 and SMURF2, an E3 ubiquitin-protein ligase, and promotes the accumulation of cytoplasmic SMURF2, thus blocking the TGFβ/SMAD pathway by facilitating SMURF2-mediated polyubiquitination and the degradation of TGFβR1 [115]. TGFβ-induced lncRNA LETS1 promotes TGFβ/SMAD signaling to form a positive feedback regulatory loop [116]. LETS1 blocks the inhibitory effect of SMAD7 on TGFβR1 by increasing the expression of NR4A1, a protein involved in SMAD7 polyubiquitination and degradation, facilitating TGFβ-induced EMT and cellular migration in breast and lung cancer [116]. LncRNA TGFB2-AS1 interacts with SMARCA4, a core subunit of the SWI/SMF chromatin remodeling complex, to suppress the transcription of its target genes, including TGFβ2 and SOX2, thereby inhibiting TGFβ signaling and impairing BCSC traits in TNBC [123].

In contrast to TGFβ-induced lncRNAs such as LITATS1 and LETS1, lncRNA SMASR is downregulated by TGFβ/Smad2/3 signaling, and the expression of SMASR in turn inhibits TGFβR1 expression by interacting with Smad2/3, thus suppressing TGFβ-induced EMT, as well as cellular migration and invasion in lung cancer [124]. The overexpression of linc00941 in colorectal cancer (CRC) activates EMT and facilitates tumor metastasis via TGF-β signaling [43]. Mechanistically, linc00941 stabilizes SMAD4 by competitively binding to its MH2 domain and suppressing its degradation mediated by β-TrCP, an E3 ubiquitin ligase. This activation of TGFβ/SMAD2/3 signaling promotes the expression of Twist1 as well as mesenchymal markers Fibronectin and vimentin, while suppressing the expression of epithelial markers E-cadherin and ZO-1 [43]. LncRNA Smyca functions as a scaffold to facilitate the interaction between Smad3 and Smad4, promoting TGFβ-induced metastasis, cancer stemness, and chemoresistance [47].

### 4.2. STAT3 Pathway

Signaling transducer and activator of transcription3 (STAT3) signaling acts as a crucial inducer of EMT [125]. The activation of the STAT3 pathway is triggered upon the binding of interleukin-6 (IL-6), growth factors, and hormones to the cell surface receptors (Figure 4B) [125,126]. Then, STAT3 phosphorylation occurs followed by the formation of STAT3 dimers, which translocate to the nucleus and activate the expression of EMT-TFs. LncRNAs are involved in the regulation of STAT3-mediated EMT [127]. LncRNA DLGAP1-AS1 promotes EMT and HCC progression by activating the JAK2/STAT3 pathway [128]. DLGAP1-AS1 increases cytokine IL-6 levels by sequestering miR-26a-5p and miR-26b-5p, while the activation of STAT3 signaling in turn elevates the expression of this lncRNA, forming a positive regulatory feedback loop. LncRNA KIAA0087 is downregulated in osteosarcoma and suppresses EMT, as well as the growth and migration of cancer cells [129]. Mechanistically, KIAA0087 acts as a ceRNA for miR-411-3p, and inhibits STAT3 signaling by upregulating the expression of suppressor of cytokine signaling 1 (SOCS1). LncRNA PVT1 induces aggressive vasculogenic mimicry formation by activating the STAT3/Slug axis and EMT in gastric cancer [130]. Specifically, PVT1 interacts with activated STAT3 proteins and facilitates the recruitment of STAT3 to the *Slug* promoter, resulting in elevated Slug expression [130]. LncRNA NEAT1 also enhances STAT3-induced EMT and metastasis in osteosarcoma through the miR-483/STAT3 axis [131].

### 4.3. Wnt Pathway

The canonical Wingless-integrated (Wnt) signaling pathway plays a key role in the activation of EMT. Wnt ligands bind to Frizzled family receptors to trigger the activation of the Wnt pathway, leading to the release of β-catenin from a complex containing APC, AXIN, and GSK3β proteins (Figure 4C) [126]. Then, the β-catenin protein is transported into the nucleus and regulates the expression of EMT-associated genes by interacting with their transcriptional coactivators, such as T cell factor (TCF) and lymphoid enhancer-binding factor (LEF) and BCL9 [126]. LncRNAs participate in the regulation of EMT induced by Wnt signaling. LncRNA SNHG10 activates Wnt/β-catenin signaling via miR-182-5p/FZD3 axis and promotes cellular proliferation and invasion in osteosarcoma [132]. Dai et al. reported an exosomal lncRNA, DOCK9-AS2, which is derived from the CSCs in papillary thyroid cancer (PTC) and promotes EMT, cellular proliferation, migration, and stemness [133]. DOCK9-AS2 upregulates the expression of β-catenin by interacting with the transcription factors SP1 and sponging miR-1972, thus inducing the activation of the Wnt/β-catenin pathway in PTC. LncRNA PKMYT1AR also promotes EMT and tumor progression in NSCLC by increasing the levels of β-catenin proteins [81]. PKMYT1AR acts as a ceRNA for miR-485-5p to upregulate the expression of PKMYT1, thus blocking the β-TrCP1-mediated ubiquitination and degradation of β-catenin. LncRNA CEBPA-DT has been reported to serve as an upstream regulator of β-catenin and to facilitate EMT and tumor metastasis in HCC via activation of Wnt signaling [134]. CEBPA-DT increases DDR2 mRNA stability by binding with hnRNPC and promoting the interaction between hnRNPC and DDR2 mRNA. Then, the increased DDR2 interacts with β-catenin and facilitates its nuclear translocation, thus upregulating Snail expression and inducing EMT [134]. LncRNA MIR100HG functions as a positive regulator of Wnt/β-catenin signalin, and promotes EMT, metastasis, and cetuximab resistance in CRC [135]. MIR100HG increases the mRNA stability of TCF7L2, a transcriptional coactivator of β-catenin, by interacting directly with hnRNPA2B1, an m6A reader, which recognizes the m6A site of TCF7L2 mRNA. Furthermore, MIR100HG is in turn upregulated by TCF7L2, forming a positive feedback loop for the regulation of the Wnt pathway [135]. LncRNA JPX promotes EMT and cell invasion in lung cancer [44]. Mechanistically, JPX upregulates the expression of TWIST1 by sponging miR-33a-5p, and the activated JPX/miRNA-33a-5p/TWIST1 axis regulates EMT by activating Wnt signaling [44].

### 4.4. Other Signaling Pathways

LncRNAs also regulate EMT via other signaling pathways [136,137]. Ref. [136] reported that lncRNA PAX-interacting protein 1-antisense RNA 1 (PAXIP1-AS1) is repressed by homeobox D9 (HOXD9), while PAXIP1-AS1 overexpression attenuates HOXD9-enhanced EMT, invasion, and metastasis in GC [136]. LncRNA GAEA is induced by increased levels of glucose as well as growth factors, such as TGFβ and IL-6, and promotes EMT [137]. GAEA interacts with MEX3C, an RNA-binding E3 ligase, to promote MEX3C-mediated K27-linked PTEN polyubiquitination, endowing PTEN with serine/threonine phosphatase activity. Then, PTEN induces the dephosphorylation of several EMT-associated proteins, including SNAI1, TWIST1, and YAP1, and facilitates the stability of these proteins [137]. Su et al. identified linc01089 as a positive regulator of EMT, which facilitates tumor metastasis in hepatocellular carcinoma (HCC) [3]. Linc01089 decreases the stability of DIAPH3 mRNA by binding to hnRNPM and facilitating hnRNPM-induced skipping of DIAPH3 exon3 during pre-mRNA splicing. By reducing DIAPH3 expression, linc01089 promotes the ERK pathway and EMT in HCC [3]. Linc01426 is also shown to promote EMT, cellular migration, and stemness in lung adenocarcinoma (LUAD) via activation of the hedgehog pathway [138]. By interacting with USP22 to facilitate USP22-mediated deubiquitination of SHH protein, Linc01426 promotes SHH stability to enhance hedgehog signaling [138]. In addition, lncTCL6 suppresses EMT and kidney cancer progression via the AKT pathway [139]. LncTCL6 recruits STAU1 protein to Src mRNA and suppresses Src expression by inducing STAU1-mediated mRNA degradation. Src protein has been reported to regulate AKT-mediated EMT [140]. Therefore, lncTCL6 regulates EMT via the Scr/AKT axis [139]. LncRNA NR2F1-AS1 (NAS1) facilitates breast cancer lung metastatic dormancy by regulating EMT and BCSC plasticity [141]. Mechanistically, NAS1 binds to NR2F1 mRNA and promotes its translation by recruiting the RNA-binding protein PTBP1, thereby leading to NR2F1-mediated transcriptional suppression of ΔNp63, which induces EMT and promotes dormancy of cancer cells in the lungs [141]. Typical lncRNAs regulating EMT, cancer stemness, and tumor development identified in various tissue malignancies are outlined in Table 1.

## 5. Clinical Implications of lncRNAs in Cancer

Numerous studies have demonstrated that lncRNAs are dysregulated in various carcinomas and their expression patterns are associated with clinicopathological features of cancer patients [113,142,143]. In addition, some lncRNAs can be detected in tumor cell-derived exosomes, which increases their stability by protecting them from ribonuclease-mediated degradation [144]. Exosomal lncRNAs are involved in cancer-associated signal transduction and modulation of tumor microenvironment [145]. Since exosomal lncRNAs are more stable, readily detectable, and exhibit cancer-specific expression patterns, they have been viewed as potential noninvasive biomarkers for diagnosis and prognosis of carcinomas. For example, ref. [146] demonstrated the potential of exosomal Xist derived from blood serum as a diagnostic marker for recurrence of triple-negative breast cancer (TNBC) . Serum exosomal Xist is elevated in patients with tumor recurrence, compared with the ones without cancer relapse. In addition, exosomal Xist is decreased following the surgical excision of primary tumors but significantly increased following cancer recurrence. The level of exosomal Xist exhibits an obvious correlation with a poor overall survival in TNBC patients [146]. Exosomal lncMLETA1 derived from highly metastatic lung cancer is increased in tumor tissues, and its level is associated with poor overall survival [147]. LncMLETA1 facilitates tumor metastasis via regulating EGFR and IGF1R expression and its level in patient plasma is correlated with tumor metastasis and a higher TNM stage, indicating its potential as a prognostic biomarker for metastasis of lung cancer [147]. In addition, refs. [148,149] reported that lncRNA MALAT1 and PCA3 derived from urinary samples may serve as biomarkers for early diagnosis for prostate cancer (PCa) in the Chinese population. Recently, they established and validated a novel lncRNA assay for early diagnosis of PCa and aggressive PCa by detection of urinary exosomal MALAT1 and PCA3 in combination [150]. Moreover, this noninvasive lncRNA assay presents a better diagnostic performance than the current clinical parameters in PCa diagnosis and could help avoid many unnecessary biopsies [150].

It is well established that lncRNAs are involved in many steps of cancer progression, including metastasis [127,134], recurrence [146], and chemoresistance [135], suggesting their enormous potential as cancer therapeutic targets. For instance, the oncogenic lncRNA PVT-1 is upregulated in nasopharyngeal carcinoma (NPC), which plays an important role in the radiation resistance of NPC by activating the KAT2A acetyltransferase and stabilizing HIF-1α [151]. LncARSR is upregulated in primary renal tumor-initiating cells (T-ICs) and is associated with a poor prognosis of clear cell renal carcinoma (ccRCC) by impeding LATS1-induced YAP phosphorylation to facilitate YAP nuclear translocation [152]. LncRNA MTR100HG positively regulates EMT via the Wnt/β-catenin pathway and facilitates cetuximab resistance and metastasis in colorectal cancer (CRC) [135]. Decreased MIR100HG expression level significantly restores the sensitivity of CRC cells to cetuximab. In addition, MIR100HG expression level is significantly higher in metastatic CRC patients compared to the ones without metastasis, and MIR100HG silencing results in the inhibition of metastasis and improved survival in a CRC mouse model [135]. Similarly, lncRNA HIF1A-As2 promotes EMT and metastasis in KRAS-driven NSCLC by forming a double-positive regulatory feedback loop with the transcription factor MYC [53]. The expression of HIF1A-As2 is elevated in NSCLC, and its increased level correlates with poor prognosis. Inhibition of HIF1A-As2 suppresses tumor cell proliferation and increases tumor sensitivity to cisplatin treatment, indicating the potential therapeutic value of targeting this lncRNA in KRAS-driven NSCLC [53].

## 6. Conclusions and Future Perspectives

The role of lncRNAs in CSCs, chemoresistance, and metastasis has been widely investigated in the past decade. By regulating gene expression through interaction with DNA, RNA, and proteins, lncRNAs play key roles in the regulation of tumor EMP via diverse signaling pathways, thereby modulating cancer stemness and tumor progression. Elucidation of the molecular mechanisms underlying lncRNA-mediated EMP during tumorigenesis and progression is crucial for developing lncRNA-based novel therapeutic strategies. As lncRNAs directly or indirectly regulate many proteins that are difficult to target conventionally such as EMT-TFs and CSC factors, the development of effective approaches targeting oncogenic lncRNAs will be a promising strategy to overcome the CSC phenotype associated with therapeutic resistance and metastasis. In addition, as more and more exosomal lncRNAs displaying cancer-specific expression patterns are identified in patient blood samples, characterization of circulating (serum) lncRNA biomarkers in patients (vs. healthy) or different molecular subtypes and clinical stages will be very useful for cancer diagnosis and prognosis. Although FDA-approved therapeutics and diagnostics based on lncRNAs have not been successfully implemented in clinic so far, they may be developed as therapeutic or diagnostic agents in the future. Furthermore, the combination of therapeutic strategies targeting tumor-promoting or oncogenic lncRNAs together with traditional cancer therapies such as chemo-therapeutics and radiation may deliver strong synergy to enhance the eradication of CSCs and further improve therapeutic efficacy in metastatic or chemoresistant carcinomas.

## Figures and Tables

**Table 1:** LncRNAs associated with EMT-mediated tumor development.

lncRNA	Cancer Type	Action Mode	Targets/Interaction Partners	Dysregulation in Cancer/CSC (Up/Down)	Roles in Tumor Development	References
CEBPA-DT	HCC	Stabilize mRNA	hnRNPC/DDR2/β-catenin	up	promote EMT and metastasis	[134]
linc01287	HCC	ceRNA	miR-298/STAT3	up	promote EMT, tumor growth, and metastasis	[127]
Linc01089	HCC	Regulate alternative splicing of mRNA	E2F1/LINC01089/hnRNPM/DIAPH3	up	promote EMT and tumor metastasis	[3]
DLGAP1-AS1	HCC	ceRNA for miR-26a-5p and miR-26b-5p	(miR-26a/b-5p)/IL-6/JAK2/STAT3	up	facilitate EMT and HCC progression	[128]
HOTAIR	HCC	Interact with protein	HOTAIR/c-Met/HOTAIR	down	maintain E/M phenotype of cells to promote metastasis	[45]
SGO1-AS1	GC	Facilitate mRNA degradation	TGFβ/ZEB1/SGO1-AS1/PTBP1/TGFβ	down	prevent EMT and metastasis	[121]
PAXIP1-AS1	GC	Bind to and destabilize protein	HOXD9/PAXIP1-AS1/PABPC1/PAK1	down	suppress EMT, cell growth, and invasion	[136]
MAGI2-AS3	GC	ceRNA	BRD4/MAGI2-AS3/(miR-141/200a)/ZEB1	up	promote cell migration and invasion	[108]
LINC00501	GC	Recruit hnRNPR to slug promoter	hnRNPR/slug	up	promote EMT, metastasis, and angiogenesis	[113]
mir200CHG	GC	Interact with miR-200c to stabilize it	miR-200c	down	inhibit EMT and lymph node metastasis	[112]
linc81507	NSCLC	ceRNA for miR-199b-5p	miR-199b-5p/caveolin1/STAT3 pathway	down	suppress EMT, cell proliferation, and migration	[50]
HIF1A-As2	NSCLC	Recruit DHX9 to MYC promoter	KARS/MYC/HIF1A-As2/DHX9/MYC	up	promote EMT, cell proliferation, tumor metastasis, and cisplatin-resistance	[53]
PKMYT1AR	NSCLC	ceRNA for miR-485-5p; stabilize β-catenin	miR-485-5p/PKMYT1; β-catenin	up	promote EMT, tumor cell migration, and CSC maintenance	[81]
NEAT1	OS	ceRNA for miR-483	miR-483/NEAT1	-	promote EMT and metastasis	[131]
KIAA0087	OS	ceRNA for miR-411-3p	miR-411-3p/SOCS1/JAK2/STAT3 pathway	down	suppress EMT, cell growth, migration and invasion, and trigger cell apoptosis	[129]
SNHG10	OS	ceRNA for miR-182-5p	miR-182-5p/FZD3	up	promote the proliferation and invasion of OS cells	[132]
JPX	lung cancer	ceRNA for miR-33a-5p	miR-33a-5p/TWIST1	up	facilitate EMT and tumor growth	[44]
SMASR	lung cancer	Interact with Smad2/3 cBR1 expression	TGF-β/Smad2/3/SMASR/TGF-β/Smad pathway	down	suppress EMT, migration, and invasion of lung cancer cells	[124]
MIR100HG	CRC	Interact with hnRNPA2B1 to stabilize TCF7L2 mRNA	hnRNPA2B1/TCF7L2	-	promote EMT, tumor metastasis, and cetuximab resistance	[135]
H19	CRC	ceRNA for miR-22-3p	miR-22-3p/MMP14	-	promote EMT and cancer metastasis	[51]
LINC00941	CRC	Stabilize SMAD4 protein	SMAD4	up	activate EMT and promote CRC metastasis	[43]
SNHG8	BC	Stabilize CDH1 mRNA	ZEB1/SNHG8/CDH1	down	suppress EMT	[115]
LINC00665	CCA	ceRNA for miR-424-5p	miR-424-5p/BCL9L	up	promote EMT, stemness, migration, invasion, and gemcitabine resistance	[142]
lncARSR	BCa	ceRNA for miR-129-5p	miR-129-5p/SOX4	up	enhance EMT, proliferation, migration, and invasion of Bca cells	[143]
ARNILA	TNBC	ceRNA for miR-204	miR-204/SOX4	-	enhance EMT and metastasis	[82]
LINC01426	LUAD	Interact with USP22 to stabilize SHH protein	USP22/SHH	up	enhance EMT, stemness, and migration	[138]
NORAD	PC	ceRNA for hsa-miR-125a-3p	hsa-miR-125a-3p/RhoA	up	enhance the hypoxia-induced EMT and promote metastasis	[83]
GAS5	PC	ceRNA for miR-221	miR-221/SOCS3	Down	suppress EMT, stemness, migration, and gemcitabine resistance	[84]
DOCK9-AS2	PTC	Interact with SP1 and act as ceRNA for miR-1972	miR-1972/CTNNB1/Wnt/β-catenin	up	promote EMT, stemness, proliferation, migration, and invasion	[133]
HOTTIP	PDAC	Interact with WDR5 to promote HOXA9 expression	HOTTIP/WDR5/HOXA9/Wnt pathway	up	promote EMT, stemness, and cancer progression	[106]
SOX2OT	Bladder cancer	ceRNA for miR-200c	miR-200c/SOX2	up	promote EMT, migration, invasion, and stemness phenotype of CSCs	[111]

Abbreviations: BC: breast cancer; Bca: bladder cancer; CCA: cholangiocarcinoma; CRC: colorectal cancer; GC: gastric cancer; HCC: hepatocellular carcinoma; LUAD: lung adenocarcinoma; NSCLC: non-small cell lung cancer; OS: osteosarcoma; PC: pancreatic cancer; PDAC: pancreatic ductal adenocarcinoma; PTC: papillary thyroid carcinoma; TNBC: triple-negative breast cancer.

## Data Availability

No new data were created or analyzed in this study.
